# Brain–computer interface game applications for combined neurofeedback and biofeedback treatment for children on the autism spectrum

**DOI:** 10.3389/fneng.2014.00021

**Published:** 2014-07-03

**Authors:** Elisabeth V. C. Friedrich, Neil Suttie, Aparajithan Sivanathan, Theodore Lim, Sandy Louchart, Jaime A. Pineda

**Affiliations:** ^1^Department of Cognitive Science, University of California, San DiegoLa Jolla, CA, USA; ^2^School of Mathematical and Computer Sciences, Heriot-Watt UniversityEdinburgh, UK; ^3^School of Engineering and Physical Science, Heriot-Watt UniversityEdinburgh, UK

**Keywords:** autism spectrum disorder (ASD), brain–computer interface (BCI), neurofeedback and biofeedback training, games, mirror neuron system, mu rhythm, heart rate variability, social engagement system

## Abstract

Individuals with autism spectrum disorder (ASD) show deficits in social and communicative skills, including imitation, empathy, and shared attention, as well as restricted interests and repetitive patterns of behaviors. Evidence for and against the idea that dysfunctions in the mirror neuron system are involved in imitation and could be one underlying cause for ASD is discussed in this review. Neurofeedback interventions have reduced symptoms in children with ASD by self-regulation of brain rhythms. However, cortical deficiencies are not the only cause of these symptoms. Peripheral physiological activity, such as the heart rate and its variability, is closely linked to neurophysiological signals and associated with social engagement. Therefore, a combined approach targeting the interplay between brain, body, and behavior could be more effective. Brain–computer interface applications for combined neurofeedback and biofeedback treatment for children with ASD are currently nonexistent. To facilitate their use, we have designed an innovative game that includes social interactions and provides neural- and body-based feedback that corresponds directly to the underlying significance of the trained signals as well as to the behavior that is reinforced.

## NEUROETIOLOGY OF AUTISM SPECTRUM DISORDER (ASD)

Autism spectrum disorder (ASD) is an increasingly prevalent condition in the U.S. with core deficits in the unique domain of human social behaviors (American Psychiatric Association, 2000; Hansen et al., 2008; Rice, 2011). Individuals with high functioning ASD show deficits primarily in social and communicative skills such as imitation, empathy, and shared attention, as well as restricted interests and repetitive patterns of behaviors. These deficits substantially impair satisfactory social interactions and prevent children from establishing adequate relations with their family or friends from their early years.

**FIGURE 1:**
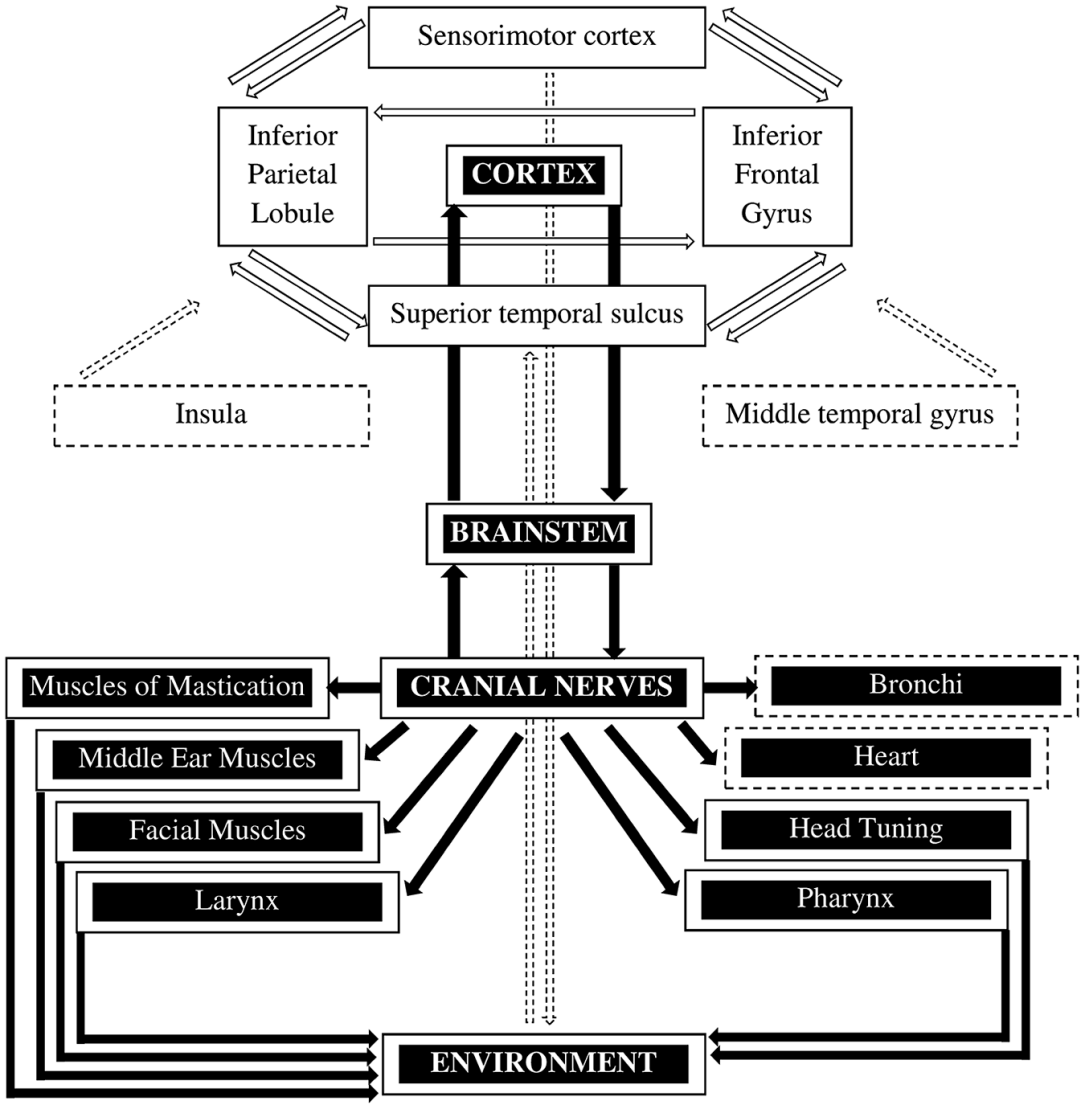
**The mirror neuron system and the social engagement system.** The mirror neuron system (MNS) is represented by the white boxes with the black letters (adapted from Pineda, 2008). The core MNS (i.e., parietal frontal in the rostral cortical convexity of the inferior parietal lobule, ventral premotor area of the inferior frontal gyrus, and the superior temporal sulcus) is extended by the sensorimotor cortex as well as the insula and the middle temporal gyrus. The MNS is involved in perceiving sensory input as well as in motor output through various processes. The social engagement system is represented by the black boxes with the white letters and shows how social communication is regulated by the cortex (adapted from Porges, 2007, 2003). The solid lines indicate the somatomotor components that control the muscles of the face and the head. The dashed lines represent the visceromotor component which consists of the myelinated vagus that controls the heart and bronchi.

To date, no single explanation can account for the broad and varied profile of the deficits in ASD. Nonetheless, exploring the neuroetiology of this disorder is a focus of our research which was prompted by the discovery of mirror neurons. The discovery of these visuomotor cells in monkey prefrontal cortex (di Pellegrino et al., 1992) and the description of a similar network of areas in the human brain, or mirror neuron system (MNS, **Figure 1**; Rizzolatti and Craighero, 2004), has provided a testable neurobiological substrate for understanding many key concepts in human social and emotional cognition directly relevant to the behavioral and cognitive deficits observed in children with ASD (Williams et al., 2001). ASD is marked by impairments in social skills - from joint attention and the ability to comprehend actions, to learning through imitation to understanding the intentions of others (Carpenter et al., 1998; Baron-Cohen, 2009). An increasing amount of studies suggest that a dysfunction in the human MNS contributes to these kinds of social deficits (Nishitani et al., 2004; Oberman et al., 2005; Théoret et al., 2005; Dapretto et al., 2006; Hadjikhani et al., 2006; Bernier et al., 2007). Specifically, deficits are likely to arise from an inability to “form and coordinate social representations of self and others” “via amodal or cross-modal representation processes” (Rogers and Pennington, 1991), the type of function ascribed to mirror neurons. However, the theory of MNS is the object of critical debates (Enticott et al., 2013). An alternative explanation, for example, is that dyspraxia rather than the MNS could account for imitation deficits in children with ASD (Mostofsky et al., 2006; Stieglitz Ham et al., 2011). Moreover, questions have been raised as to whether the discovery of mirror neurons in monkeys can be translated to explaining human social behavior (Hickok, 2009; Turella et al., 2009).

From an anatomical perspective, an *underconnectivity* hypothesis has been proposed by Just et al. (2004), which posits that “autism is a cognitive and neurobiological disorder marked and caused by underfunctioning integrative circuitry that results in a deficit of integration of information at the neural and cognitive levels.” Reduced connectivity, especially in ASD individuals, is consistent across studies using various cognitive, emotional, and social tasks (Villalobos et al., 2005; Welchew et al., 2005; Just and Varma, 2007) and in both default mode and task-related functional connectivity magnetic resonance imaging (fcMRI) studies. While a general theory of disordered connectivity has emerged, the nature of the disorder is not yet clear. To bring some level of reconciliation along various observations, several investigators have proposed a compromise solution that focuses on both local overconnectivity and long range underconnectivity (Anderson et al., 2011). This is not inconsistent with the MNS hypothesis since over- and under connectivity likely characterizes this specific network.

From electrophysiological studies of ASD, there is an equally emergent framework. Using phase coherence in multiple frequency bands as a measure of functional connectivity, evidence shows both global hypoconnectivity and local hyperconnectivity (Murias et al., 2007). Specifically, locally elevated coherence in the theta (3–6 Hz) frequency range in ASD subjects, particularly over left frontal and temporal regions, as well as globally lower coherence in the lower alpha range (8–10 Hz) within frontal regions was found (Murias et al., 2007). In contrast, decreased local and decreased, as well as increased, long range spectral coherences for the ASD-group in comparison to controls was reported recently (Duffy and Als, 2012). Furthermore, the coherence patterns in the ASD group were unusually stable across a wide spectral range, which was interpreted as “over-damped neural networks.” Other studies have reported lower delta and theta coherences within as well as between hemispheres across the frontal region, with delta, theta, and alpha hypocoherences over temporal regions while in posterior regions, low delta, theta, and beta coherences were observed (Coben et al., 2008). Moreover, increased gamma activity over parietal cortex (Brown et al., 2005), decreased left hemispheric gamma power (Wilson et al., 2007) and increased connectivity of temporal lobes with other lobes in the gamma frequency band (Sheikhani et al., 2012) have been reported for individuals with autism. Based on the neuroanatomical, functional, and electrophysiological evidence, we hypothesize that a range of over- and underconnectivity in children with ASD, particularly in the MNS system, correlates with levels of performance in cognitive, emotional, and behavioral outcomes.

## RATIONALE FOR BRAIN–COMPUTER INTERFACE (BCI) AND NEUROFEEDBACK TRAINING (NFT) FOR ASD

**FIGURE 2:**
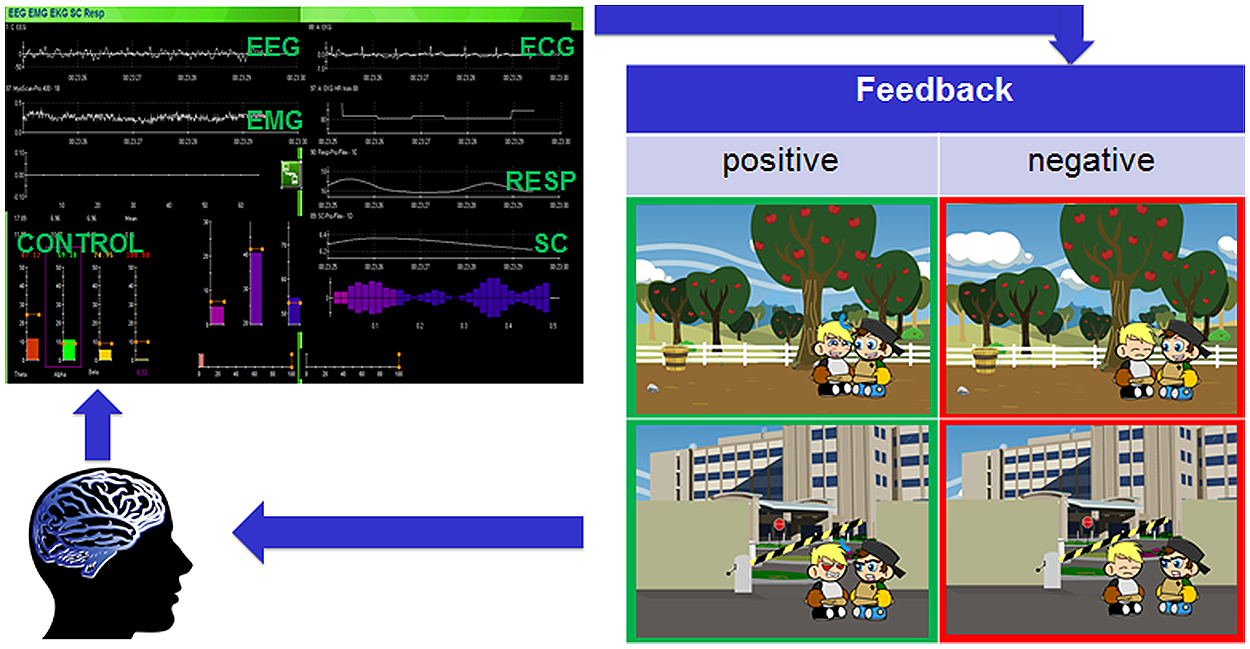
**Closed feedback loop of the Social Mirroring Game.** The user’s EEG and peripheral physiological measures are recorded (Thought Technology Ltd., Canada) and fed into the Social Mirroring Game which gives the user visual feedback. For positive feedback (i.e., indicated in green), the child’s avatar must first approach the non-player character (NPC) and while facing him, the player has to show appropriate brain and/or peripheral physiological activity. The rewarding feedback involves the child’s avatar imitating the facial emotions of the NPC. The negative feedback (i.e., indicated in red) involves the child’s avatar being not responsive to the NPC. By means of the feedback, the user can learn to change his/her brain activity voluntarily and thus can control the game.

We have previously hypothesized that BCI-based neurofeedback using specific electroencephalographic (EEG) frequency bands should induce neuroplastic changes and lead to normalization of the MNS (Pineda et al., 2012). A BCI allows real-time information of brain activity to be fed back to a user by means of a computer in a closed loop (**Figure 2**) enabling control and natural operation of brain oscillations across cortical networks *in vivo* and in near real time (Nowlis and Kamiya, 1970; Wolpaw et al., 2002; Friedrich et al., 2009, 2013; Neuper et al., 2009; McFarland et al., 2010). The possibility of volitional control of these oscillations suggests – provided that they play a causal role in specific cognitive functions – that it is theoretically plausible that their modulation can have a functional impact.

The gold standard of neurofeedback training (NFT) is based on quantitative electroencephalography (QEEG). This approach is able to identify unique electrophysiological phenotypes (Coben et al., 2010), which makes the possibility of a QEEG-based NFT as a personalized therapeutic approach viable. That approach improves the likelihood that the intervention will be effective by first identifying activity at specific electrode sites that are outside the norm, i.e., comparing the data to already existing normative databases, and then targeting the sites of greatest difference for NFT (Cantor and Chabot, 2009; Coben and Myers, 2010; Thompson et al., 2010). Recent QEEG guided studies have reported behavioral improvements on a number of measures and it has been used to achieve behavioral and neuroregulatory improvements, primarily in children with attention deficit hyperactivity disorder, but also in those with ASD (Coben and Myers, 2010; Thompson et al., 2010). More specifically, assessment guided NFT was used to reduce hyperconnectivity in posterior-frontal to anterior-temporal regions (Coben and Padolsky, 2007). Following NFT, parents reported symptom improvement in 89% of the experimental group, with very little change in the control group. Improvement also occurred in the areas of attention, visual perceptual functioning, language, and executive functioning, with a 40% reduction in core ASD symptoms as assessed by the Autism Treatment Evaluation Checklist. There was also decreased hypercoherence in 76% of the experimental group as measured by a post-training QEEG. Kouijzer et al. (2009b) reported improved executive functions for attention control, cognitive flexibility, and planning as well as improved social behavior after a theta/beta-based NFT training in children with ASD compared to a waiting list group. The linear decrease in theta power and the increase in low beta power were hypothesized to enhance activation of the anterior cingulate cortex, which has been found to show reduced connectivity in ASD individuals (Cherkassky et al., 2006). A follow-up after twelve months revealed maintenance of the described outcomes on both executive functioning and social behavior, suggesting that NFT treatment can have long-term effects (Kouijzer et al., 2009a). The examination of physiological and behavioral data from the children themselves as well as the use of a control group and the comparison between different NFT paradigms (i.e., increase/decrease of different EEG rhythms) or between different electrode sites (i.e., occipital versus central) is crucial as parents’ evaluations could be biased.

In addition to the above discussed promising NFT paradigms, research in our laboratory focus on training children on the spectrum to modulate their mu rhythm. Pineda et al. (2008, 2014) reported improvements in symptoms of autism evaluated by the parents as well as normal mu suppression after a mu-based NFT in contrast to a control group. Several studies from different laboratories have shown that mu rhythm phenomenology (alpha range: 8–13 Hz; beta range: 15–25 Hz) is linked to mirror neuron activity in that both are sensitive to movement, as well as to motor, affective, and cognitive imagery (Hari et al., 1997; Klimesch et al., 1997; Pfurtscheller et al., 1997, 2000; Muthukumaraswamy et al., 2004; Oberman et al., 2005; Pineda et al., 2008; Keuken et al., 2011). Furthermore, it was reported that mu rhythms, like mirror neurons, are modulated by object-directed actions (Muthukumaraswamy and Johnson, 2004; Muthukumaraswamy et al., 2004) and that during self-initiated, observed, and even imagined movement, mirror neuron asynchrony results in mu rhythm suppression (Pineda et al., 2000; Pineda, 2005; Neuper et al., 2009). Recently, it was demonstrated that mu rhythm suppression to movement observation is dependent on whether someone wants to be socially involved with another person and on the kind of movement observed (i.e., kinematic or goal-relevant; Aragón et al., 2013).

Both, functional magnetic resonance imaging (fMRI) and (EEG) techniques have demonstrated that mu rhythm suppression occurs in human MNS regions during tasks that activate this system, namely the inferior parietal lobe, dorsal premotor cortex, and primary somatosensory cortex (Arnstein et al., 2011). In individuals with autism, this mu rhythm suppression is not observed compared to typically developing children, supporting the role of an altered MNS (Oberman et al., 2005, 2008; Bernier et al., 2007; Oberman and Ramachandran, 2007). In contrast, Raymaekers et al. (2009) did not find a difference in mu suppression to self-executed or observed movement in autistic individuals in comparison to controls. Braadbaart et al. (2013), Arnstein et al. (2011) explained the reduced mu rhythm suppression in ASD as a more general deficit in visuomotor integration although they confirmed the relationship between mu rhythm suppression and the activation of mirror neuron areas described. In summary, although there is a lack of consensus, the majority of the literature provides enough evidence to speculate that training children to control mu rhythms may lead to functional improvements.

## THE POLYVAGAL THEORY: A RATIONALE FOR COMBINING NFT AND BIOFEEDBACK FOR ASD

Cortical deficiencies might not be the only cause of ASD symptoms. Individuals with ASD show deficits in emotional responsiveness (Scambler et al., 2007). This phenomenon cannot be solely explained by specific cortical deficiencies but likely involves peripheral physiological reactions of the autonomous nervous system (Thompson and Thompson, 2009; Thompson et al., 2010). The Polyvagal Theory proposed by Porges (2003, 2007) links cortical and peripheral physiological components in the social engagement system, which is responsible for facial expression, head turning, vocalization, listening, and other socially relevant behaviors that are atypical in individuals with ASD (**Figure 1**). According to this theory, autism is associated with autonomic states that foster the misinterpretation of a neutral environment as being threatening, and consequently can change normal vagal activity and result in withdrawal from social interaction. Thus, individuals with ASD show deficits in cardiac vagal tone regulation and impaired heart rate reactivity to external stimuli (i.e., heart rate variability, HRV), which are linked to the social engagement system (Porges, 2003). Consistent with this, Thayer and Lane (2000) suggested a model of neurovisceral integration, which proposes that HRV is an index of individual differences in regulated emotional responding (Appelhans and Luecken, 2006). Moreover, recent publications argue that heart rate and its variability play an important role in emotion recognition (Quintana et al., 2012) as well as for BCI control (Kaufmann et al., 2012; Pfurtscheller et al., 2013). This suggests that training children on the spectrum to increase their vagal tone via biofeedback (Lehrer, 2007; Gevirtz, 2010, 2007) should lead to additional improvements in the social engagement system, including emotional responsiveness.

In contrast to vagal tone, which is an indicator of parasympathetic activity (Task, 1996), skin conductance is a reliable index for sympathetic arousal of the autonomous nervous system (Bach et al., 2010). While different patterns of skin conductance in individuals with ASD have been shown (Schoen et al., 2008), it is not yet clear what kind of differences occur in skin conductance and heart rate between individuals with ASD and controls (Levine et al., 2012; Mathersul et al., 2013). Therefore, more research including peripheral physiological parameters in individuals with ASD is crucial to develop a more comprehensive model of the disorder and thus produce better treatment approaches.

## GAME APPLICATIONS TO COMBINE NFT AND BIOFEEDBACK INTRODUCING A NOVEL GAME PLATFORM FOR CHILDREN ON THE SPECTRUM

One treatment approach is to combine biofeedback of peripheral physiological reactions with neurofeedback of cortical electrophysiology and to do this in the context of play. Play is an ideal medium to engage children and help develop their motor skills, communication, problem solving and social skills (Oden and Asher, 1977; Hughes, 1998; MacDonald et al., 2013). There are many challenges in creating NFT and biofeedback games, not least the application must maintain player interest (Tan and Jansz, 2008) and secondly the limited genres available for ASD. The visualization of the feedback in NFT and biofeedback paradigms ranges from controlling a simple bar graph to more sophisticated visual renditions. However, the feedback typically is not related to the specific significance of the signals being trained or the anticipated behavioral changes. For example, the feedback might be the speed or response of a race car – indicating the level of control of the mu rhythm – while the anticipated outcome is that training the mu rhythm will lead to better imitation behavior. However, a specific feedback (i.e., showing the control of imitation behavior instead of a race car on the screen) for specific signals being trained (i.e., training the mu rhythm to improve imitation behavior) might be more effective in linking brain activation and anticipated behavior. Accordingly, training the EEG mu rhythm as well as training HRV should increase positive social behavior in children with ASD. Investigating this research question requires the development and implementation of a game platform that includes social interactions and specific feedback based on imitation behavior and emotional responsiveness. Therefore, we propose games such as the newly developed Social Mirroring Game (**Figure 2**), which requires children with ASD to modulate their brain activity (i.e., mu power) and/or peripheral physiological activation (e.g., increase in vagal tone) in gaming parts as well as in social situations between the child’s avatar and his friend (i.e., a non-player character, NPC) in order to get rewarded. The rewarding feedback involves the child’s avatar imitating the facial emotions of the NPC. The role-playing game mechanics allow the temporal dynamics of the player to be recorded to track behavior changes, accommodate game mechanic changes and to help direct the player.

For a game with the goal of improving social interactions, it is important to address the following questions: (1) is playing a social game without modulating physiological activity able to enhance appropriate social interactions? (2) is a single-person game rather antisocial than promoting social behavior? and (3) can the learned behavior be transferred from the gaming situations to the real-world?

First, it has been shown that role-play mechanism is a powerful tool towards assessing and intervening on social behaviors. Without actually manipulating brain or peripheral physiological activity, the Fearnot! social agent demonstrator (Aylett et al., 2006; Enz et al., 2008) was successful in proving that game-based platforms could have significant effect on a children population in domains related to social behavior (i.e., anti-bullying). Moreover, playing a cooperative computer game was shown to reinforce social interactions and appropriate social communicative behavior in children with ASD (Piper et al., 2006). MOSOCO (Escobedo et al., 2012) is a mobile augmented reality application based on the Social Compass curriculum (Tentori and Hayes, 2010) that facilitates practicing and learning social skills in children with ASD in social groups of neurotypical children. The results indicate that such assistive technologies with game-like interactions and role-play where points and rewards are earned improve the learning experience.

Second, the Social Motivation Adaptive Reality Treatment Games (SMART-Games; Gotsis et al., 2010) address the issue of single-player versus multiplayer games by using an avatar that exhibits different moods as an interface to a computer game which can be played as single-player, virtual or co-located multiplayer. The ECHOES project (Bernardini et al., 2014, 2012) dispels the myth that single-person games are inherently antisocial as it increases social interactions in the real world for some children with ASD.

Third, the ECHOES project also illustrates how role-play mechanics transferred to a virtual agent can be used to increase learned social skills from the game to the real world for children with ASD. The ECHOES game world, however, has no capability to adapt as its behavioral agent does not take psychophysiological inputs from a player. It is likely that brain and peripheral physiological activity is different in the video-game scenario compared to face-to-face interaction with a peer in real-life and the generalization has yet to be shown. However, like Fearnot!, ECHOES demonstrated the benefits of a game-based intervention towards social understanding, mechanisms and behavioral regulation in social situations.

In summary, games such as the Social Mirror Game are moving in the right direction and are promising tools to examine and improve the effects of training physiological measurements during social and emotional imitation behavior and interactions.

## CONCLUSION

This review highlights the importance of using BCI, NFT, and biofeedback to provide novel insights about the physiological correlates of ASD, as well as the need to design innovative treatment approaches for such individuals. To date, the complex mechanisms underlying autism are not entirely understood. We propose that combining NFT and biofeedback may prove to be more effective than traditional approaches and describe a new game interface designed specifically for this purpose, i.e., to link appropriate behavior, neurophysiological and peripheral physiological reactions in social situations. As the rewarding feedback corresponds directly to the underlying significance of the signals we train as well as to the behavior we aim to reinforce and through the reinforcement of all facets of social interactions, substantial improvements in behavior, cognition and emotion can be expected for children with ASD.

## Conflict of Interest Statement

The authors declare that the research was conducted in the absence of any commercial or financial relationships that could be construed as a potential conflict of interest.
